# The complete nucleotide sequence of *Viburnum odoratissimum* chloroplast genome

**DOI:** 10.1080/23802359.2020.1749151

**Published:** 2022-04-11

**Authors:** Le Thi Yen, Joonho Park

**Affiliations:** Department of Fine Chemistry, Seoul National University of Science and Technology, Seoul, South Korea

**Keywords:** Chloroplast genome, *Viburnum odoratissimum*, phylogenetic tree

## Abstract

The complete chloroplast genome (cpDNA) *Viburnum odoratissimum*is sequenced and assembled from the whole genome data. The cpDNA of *V. odoratissimum* is 158,744bp in lengthwith the overall GC content of 38.1%. It consists of a pair of inverted repeats (IRa and IRb, 26,494bp) which separate a large single copy (LCS, 87,348 bp) and small single copy (SSC,18,408 bp). The complete chloroplast genome contains 129 genes, including 84 protein-coding genes, 8 rRNA genes and 37 tRNA genes. A phylogenetic tree of DNA sequences of barcoding regions, including *rbcL, matK, psbA-trnH*from 8 species of the Genus *Viburnum* shows that *V. odoratissimum* is closely related to *V. furcatum* and *V. burejaeticum.*

Chloroplast is an essential organelle known as plastid that performs photosynthesis, where the light energy is converted into chemical energy. Chloroplasts contain own genome inherited fromcyanobacteria endosymbionts, which distinct from the nuclear genome (Tong et al. 2016). The chloroplast genomes (cpDNA) area circular DNA highly conserved among land plants than nuclear and mitochondrial genomes (Supriya and Mallinath Priyadarshan 2019; Jansen et al. 2005). The size of cpDNA varies within 120–160kb in land plants with a quadripartite structure consisting of a large single copy (LSC) and small single copy (SSC), partitioned by a pair of inverted repeat regions (IRa and IRb)(Wang et al. 2018).The cpDNA was found shared 81% of genes with their ancient algae species and typically contained four copies of rRNA genes, a number of tRNA genes and some protein-coding genes (Jiao and Guo 2014).

The Dipsacales is a branch within Asteridae clade and contains over 1000 species (Bremer et al. 2002). The order includes two families, Adoxaceae and Caprifoliaceae under a classification of the APG III system of 2009. Morphological characters and molecular datasets analyses divideAdoxaceae into *Viburnum*, *Sambucus*, and *Adoxina* which contains *Adoxa*, *Tetradoxa* and *Sinadoxa* (Donoghue et al., n.d.).*Viburnum*is themost basal lineagein Adoxacae family (Jacobs et al. 2010). Therefore, the complete cpDNA of Viburnum would provide critical insights into the ecological and evolutionary relationships of Adoxaceae. *V.odoratissimum*is a well-known evergreen shrub distributed in Asia. It possesses excellent medicinal properties used as traditional medicine for menstrual, stomach, and kidney cramps (Ge et al. 2018).

*V.odoratissimum*tissues was collected from Jeju island, Korea. The specimen was stored at National Institue of Biological Resouces, Korea.The whole genomic DNA was extracted using DNeasy Plant Mini Kit (Qiagen, Seoul, Korea).In this study, we reported the complete chloroplast genome of *V.odoratissimum* obtained from the PACBio sequencing system (Pacific Biosciences, Menlo Park, CA).

The cpDNA of *V.odoratissimum* is 158,744bp in length composed of two IR regions of 26,494 bp that divide a LSC region of 87,348 bp and a SSC region of 18,408bp. The total GC content of the *V.odoratissimum*cpDNA is 38.1% in which IR regions, LSC, SSC are 43%, 36.4% and 32.1%, respectively. The entire genome encodes for129 individual genes with 84 protein-coding genes, 8 rRNA genes and 37 tRNA genes. The IR region contains 17 duplicated genes (*rpl2, rpl23, trnM-CAU, ycf2, trnL-CAA, ndhB, rps7, rps12, rrn16, trnV-GAC, trnI-GAU, trnA-UGC, rrn23, rrn4.5, rrn5, trnR-ACG, trnN-GUU*). A total of 15 intron-containing genes is shown, 13containing one intron (*rpl2, trnK-UUU, rps16, atpF, rpoC1, trnL_UAA, trnV-UAC, ndhB, rps12, trnI-GAU, trnA-UGC, ndhA*) and two (*ycf3, clpP*)containing two introns. The complete chloroplast genome sequence was deposited in GenBank under the Accession No. MN836381.

A phylogenetic tree was constructed using DNA sequencesof *rbcL, matK, psbA-trnH*of 8 related species from NCBI database aligned with MUSCLE alignment. MEGA7 (MEGA Inc., Englewood, NJ) was performed to construct maximum-likelihood tree. *V.odoratissimum*is closely related to *V.furcatum*and *V.burejaeticum* (Figure 1).

**Figure 1.**
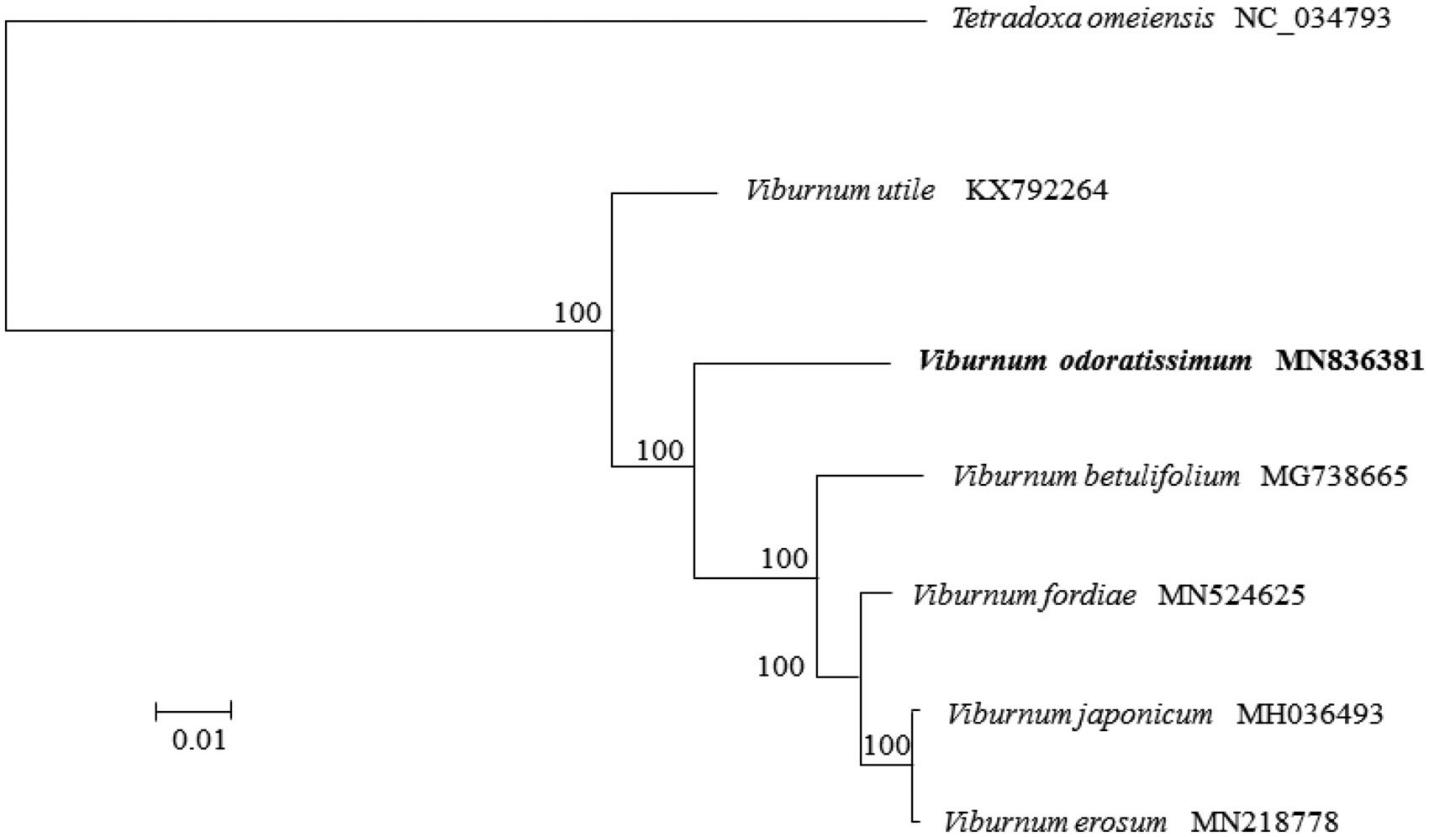
Phylogenetic tree constructed from complete genomes of 8 *Viburnum* species using maximum-likelihood analysis with 1000 boostrap replicates.
